# A Bioelectrochemical Approach to Characterize Extracellular Electron Transfer by *Synechocystis* sp. PCC6803

**DOI:** 10.1371/journal.pone.0091484

**Published:** 2014-03-17

**Authors:** Angelo Cereda, Andrew Hitchcock, Mark D. Symes, Leroy Cronin, Thomas S. Bibby, Anne K. Jones

**Affiliations:** 1 Department of Chemistry and Biochemistry, Arizona State University, Tempe, Arizona, United States of America; 2 Ocean and Earth Sciences, University of Southampton, Southampton, United Kingdom; 3 School of Chemistry, The University of Glasgow, Glasgow, United Kingdom; Texas A&M University, United States of America

## Abstract

Biophotovoltaic devices employ photosynthetic organisms at the anode of a microbial fuel cell to generate electrical power. Although a range of cyanobacteria and algae have been shown to generate photocurrent in devices of a multitude of architectures, mechanistic understanding of extracellular electron transfer by phototrophs remains minimal. Here we describe a mediatorless bioelectrochemical device to measure the electrogenic output of a planktonically grown cyanobacterium, *Synechocystis* sp. PCC6803. Light dependent production of current is measured, and its magnitude is shown to scale with microbial cell concentration and light intensity. Bioelectrochemical characterization of a *Synechocystis* mutant lacking Photosystem II demonstrates conclusively that production of the majority of photocurrent requires a functional water splitting aparatus and electrons are likely ultimately derived from water. This shows the potential of the device to rapidly and quantitatively characterize photocurrent production by genetically modified strains, an approach that can be used in future studies to delineate the mechanisms of cyanobacterial extracellular electron transport.

## Introduction

The ability of a number of microorganisms to exchange electrons with solid external substrates, a process referred to as extracellular electron transport (EET), has spawned the emerging field known as electromicrobiology and is foundational to understanding geomicrobiology. This area has attracted considerable attention for possible applications in renewable energy generation [1], [2], [3]. The most commonly described device is a microbial fuel cell (MFC), a system in which microorganisms are used as anode catalysts to oxidize an externally-provided fuel, often a component in wastewater, with concomitant production of electricity and reduction of oxygen to water at the cathode [4]. In a simple variation on this idea, electrons provided by the anode can be used by microorganisms to produce desired chemicals in the reductive reactions at the cathode, a process referred to as microbial electrosynthesis [5], [6], [7]. By utilizing photosynthetic organisms in the anode, water can be used as the electron source in a device that is referred to as a bio-photovoltaic cell (BPV) [8], [9], [10], [11], [12], [13], [14], [15]. In principle, a BPV can be used for solar-powered, CO_2_-neutral production of chemicals or electricity. However, the efficiency of these devices is very low, and mechanistic understanding of EET by phototrophs is almost nonexistent. This despite the fact that an understanding of the EET process may allow genetic engineering and synthetic biology approaches to substantially improve the power output of BPVs.

The limited mechanistic understanding of EET that exists has been developed based on studies of the chemoheterotrophic, anode-respiring bacteria of the *Shewanella* and *Geobacter* spp. The mechanisms that have been described for EET fall into two categories: direct and indirect [16]. Indirect mechanisms are those that rely on a soluble redox mediator to transfer electrons between the cell and the insoluble substrate. This mediator can be either microbially produced, such as flavins in natural systems [17], or exogenously added, such as ferricyanide, in the case of technological devices [11]. Direct mechanisms are those in which EET occurs *via* physical contact between the solid surface and the microorganism or microbial biofilm. A number of conductive microbial components have been hypothesized to facilitate this direct mechanism including conductive proteinaceous filaments known variously as conductive pili or bacterial nanowires, cell surface *c*-type cytochromes, or unknown redox active components embedded in the extracellular polysaccharide matrix [18], [19], [20], [21]. It is interesting to note that competing reports in the literature suggest that the same organism may utilize different mechanisms depending on the precise conditions of growth and measurement, further complicating the picture [22].

Light driven power output in BPVs from photosynthetic organisms including cyanobacteria [8], [9], [11], algae [10], and higher plants [23], [24], [25] has been demonstrated. The majority of this research has focused on improving current outputs either via device engineering or strain selection rather than attempting to understand or optimize the underlying biochemical processes that lead to extracellular current generation [15], [26]. Since these studies have been focused on maximal power output, the majority have used an exogenous, soluble redox mediator to shuttle electrons indirectly between the microorganism and the electrode surface [8], [11], [27], [28]. Although this approach usually results in increases of observed power, there are several disadvantages associated with using redox mediators. First, concerns regarding sustainability, cost and toxicity make the use of mediators on an industrial scale impractical [14]. Second, these mediators permeate to the cell interior and may shuttle electrons to and from a number of different, ill-defined intracellular components and pathways [28]. Thus understanding and optimizing the biochemistry in these mediated systems is exceedingly difficult. Notably, McCormick and coworkers reported the first study of pure cultures of phototrophs using a mediatorless BPV [10]. However, their work was limited by the requirement that the cells under investigation form a stable, mature biofilm at the electrode surface. This is a severe limitation since many well-studied, genetically tractable phototrophs do not fulfil this criterion and robust biofilm formation requires considerably more time than planktonic cell growth.

Herein we report the first mediatorless BPV designed to evaluate photocurrent production by the well studied model cyanobacterium *Synechocystis* sp. PCC6803 (hereafter *Synechocystis*). The electrochemical cell consists of a single chamber, potentiostatically controlled, bio-electrochemical system in which planktonically grown *Synechocystis* cells are immobilized at a carbon cloth electrode. The system generates reproducible photocurrents without addition of an exogeneous redox chemical mediator, and we show that the device can be used to measure differences in photocurrent production between wild type and mutant cells in the presence/absence of chemical inhibitors. Thus this device is suitable for quantitative screening of genetically modified strains deficient in cellular components to map the biochemical pathways thought to produce and inhibit extracellular electron transfer by cyanobacteria and other photoautotrophs.

## Results

### A mediatorless bioelectrochemical system for measuring extracellular photocurrent from *Synechocystis*

**Figure 1:**
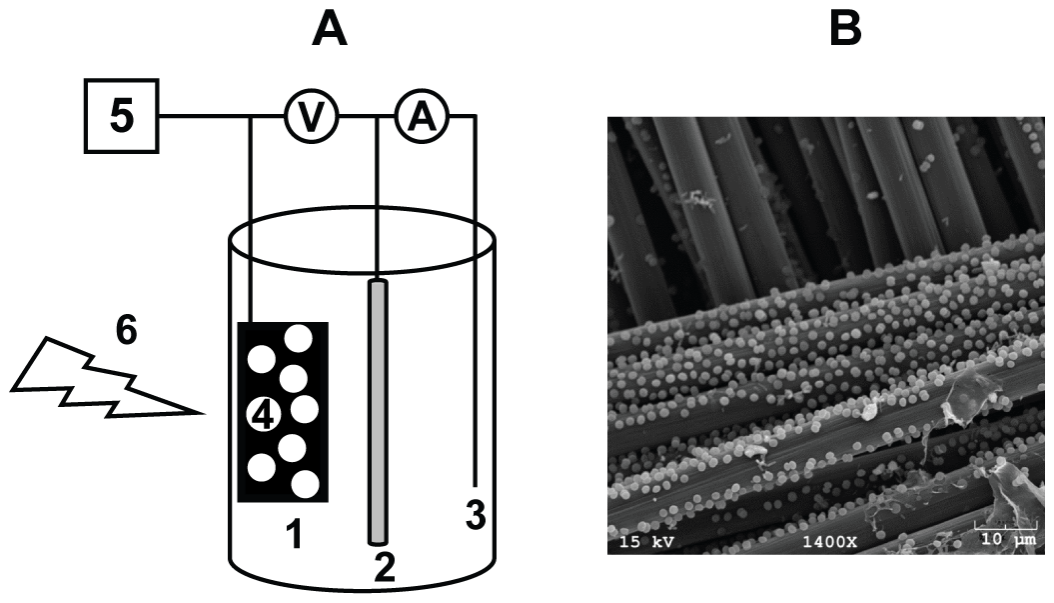
The bio-electrochemical setup used to measure photocurrent in this study. (A) Schematic representation of the bio-electrochemical device. The single chamber glass electrochemical cell contained 10 ml of BG11 as the electrolyte, a saturated Ag/AgCl reference electrode (2) and platinum wire as the counter electrode (3). *Synechocystis* cells (4) were dried onto the working carbon cloth electrode (1) and electrochemical measurements were performed using a CHI 1200A (CH instruments, Inc. Austin, Texas) potentiostat (5) with illumination provided by a red LED light source (6). (**B**) Scanning electron micrograph of wild type *Synechocystis* cells immobilized on a carbon cloth electrode.

The bio-electrochemical system developed in this work is described in the experimental procedures and depicted schematically in Figure 1A. In short, the device is an open, glass, single chamber, three-electrode electrochemical cell under potentiostatic control with a working electrode consisting of woven carbon fabric. One of the goals of this investigation was to establish a fast methodology for measuring EET that does not rely on extensive, i.e. multi-hour or day, biofilm formation.

The *Synechocystis* cells investigated in this study were grown planktonically under photoautotrophic (unless otherwise stated) conditions and harvested *via* centrifugation. For incorporation into the electrochemical device, harvested cells were resuspended in fresh BG11, diluted to the desired optical density with fresh medium and allowed to dry on the electrode surface over the course of two hours (Figure S1).

Figure 1B shows an SEM image of the *Synechocystis* cells immobilized on a carbon cloth electrode. The micrograph shows that the cells are uniformly dispersed throughout the material in a relatively dense single layer on the carbon surface. Although some may be close enough for cell-to-cell contact, the majority are isolated from adjacent cells by a distance of at least 1 µm. It is worth noting that SEM sample preparation is likely to negatively affect the number of cells attached to the electrode, and therefore the image of cells on the carbon cloth shown in Figure 1B underestimates the coverage anticipated in the electrochemical experiments. The cells are presumed to be in direct physical contact with the underlying carbon substrate, and any electrical communication may be direct or rely on a microbially secreted, redox-active small molecule. While conductive bacterial “nanowires” have been hypothesized to mediate extracellular electron transfer interactions between microbial and solid surfaces in specific cases [18], [19], such cellular appendages are not visible at the resolution used in this study.

**Figure 2:**
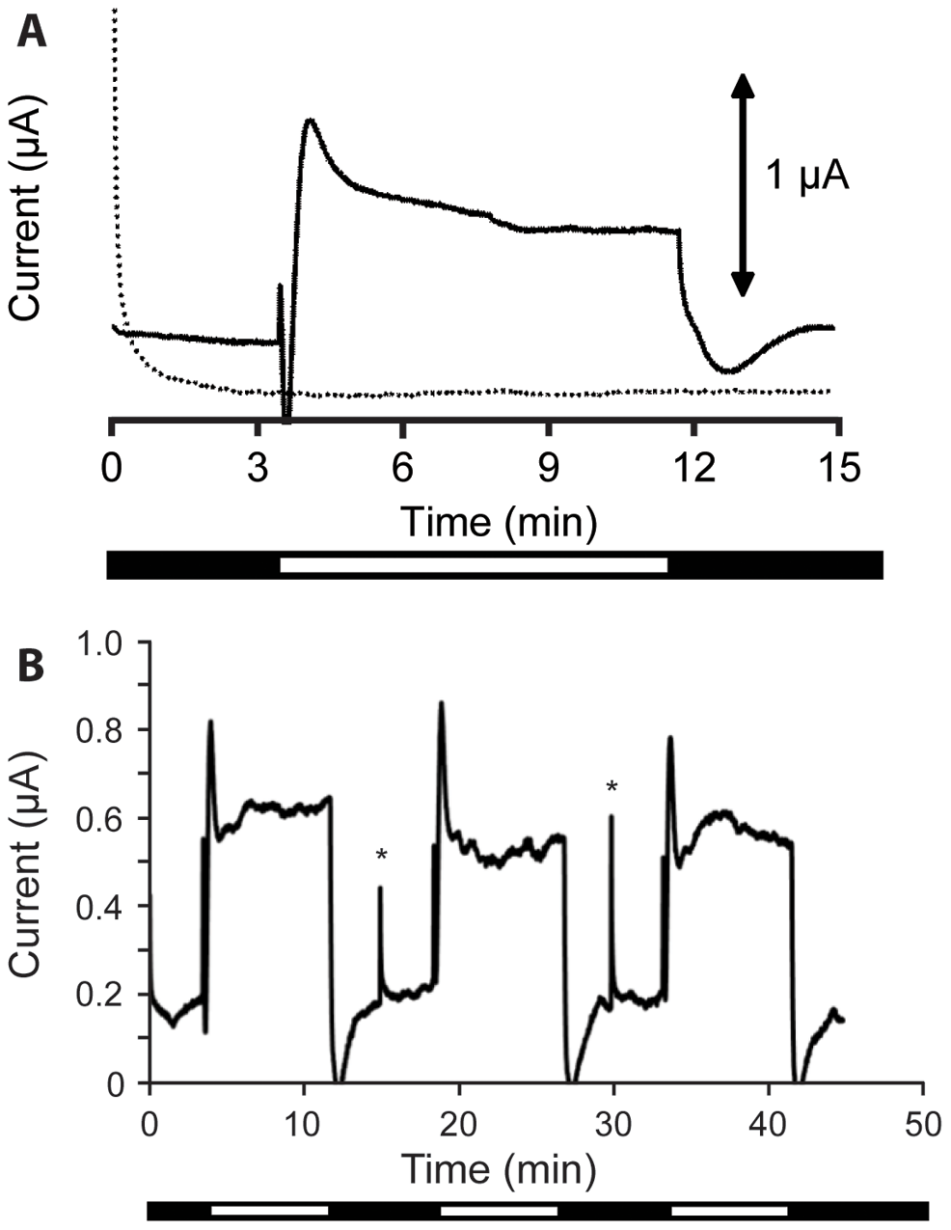
Chronoamperogram showing photocurrent produced by *Synechocystis* cells immobilized on a carbon cloth electrode. (A) The solid line shows a representative current-time trace for immobilized *Synechocystis* cells in a chronoamperometry experiment. The current output in the dark was allowed to stabilize prior to illumination, with the steady state increase in current output in the light measured as the photocurrent. Controls with heat inactivated *Synechocystis* cells (dotted line) did not produce current under dark or illuminated conditions. (B) Representative chronoamperometric trace showing photocurrent produced by *Synechocystis* exposed to three consecutive dark/light cycles. Stars mark electrical spikes caused by restarting the electrochemical equipment. For both panels, the black and white bars below the x-axis indicate periods of dark and illuminated conditions, respectively.

Extracellular electron transfer from *Synechocystsis* to the carbon electrode and its dependence on light was probed by measuring the current produced at an applied electrochemical potential of +237 mV *vs.* SHE, a potential chosen, based on the thermodynamics of photosynthetic electron transport, to be sufficiently oxidizing for cells to be able to spontaneously transfer electrons to the electrode surface. As shown in Figure 2, following pre-incubation for 15 min at the desired electrochemical potential, immobilized cells were first monitored in the dark until a stable current was attained, typically requiring approximately 4 min. Illumination of the electrochemical apparatus with red light (peak λ = 660 nm, maximum intensity = 20 W m^−2^ [110 µmol photons m^−2^ s^−1^]) then resulted in production of a positive photocurrent, the magnitude of which stabilized on the timescale of *circa* 90 seconds. A sharp reduction in current to a level comparable to that previously measured in the dark was observed when the apparatus was returned to dark conditions. The response could be reproduced with the same magnitude and time course over several cycles (at least 3, see Figure 2), indicating that the cells do not appreciably dissociate from the electrode on the timescale of the experiment and their functional integrity is not observably compromised by the measurement. Although absolute current magnitudes varied between experiments, photocurrent magnitude, defined as the difference between current measured in light and dark conditions, was reproducible in independent measurements using different batches of cells and working electrodes. With that in mind, since we are interested in evaluating electron flow arising from light-driven processes, only photocurrent will be reported for the remainder of this work. Normalizing to the area of the electrode, photocurrent values as high as 0.4 µA cm^−2^ were obtained.

To confirm that the observed photocurrent was attributable to an electron transport process requiring live cyanobacteria, a negative control experiment in which heat-killed *Synechocystis* cells were immobilized at the working electrode was performed. Electrodes treated with the dead cells produced background currents that were unchanged upon illumination (Figure 2, dotted trace). In fact, the current profile generated from heat-killed cells is analogous to the profile obtained in the absence of any biological material (Figure S2), confirming photocurrent generation is a result of one or more biological processes associated with the live cyanobacterial cells.

### Parameters determining photocurrent magnitude

To identify parameters determining the magnitude of the observed photocurrent, electrochemical experiments were performed with different quantities of cells immobilized on the electrode surface, at different light intensities, and in the presence of an exogenously provided redox mediator. For all of the following experiments, wild type cells grown under photoautotrophic conditions until stationary phase (OD_750_ = 2.0) were utilized.

**Figure 3:**
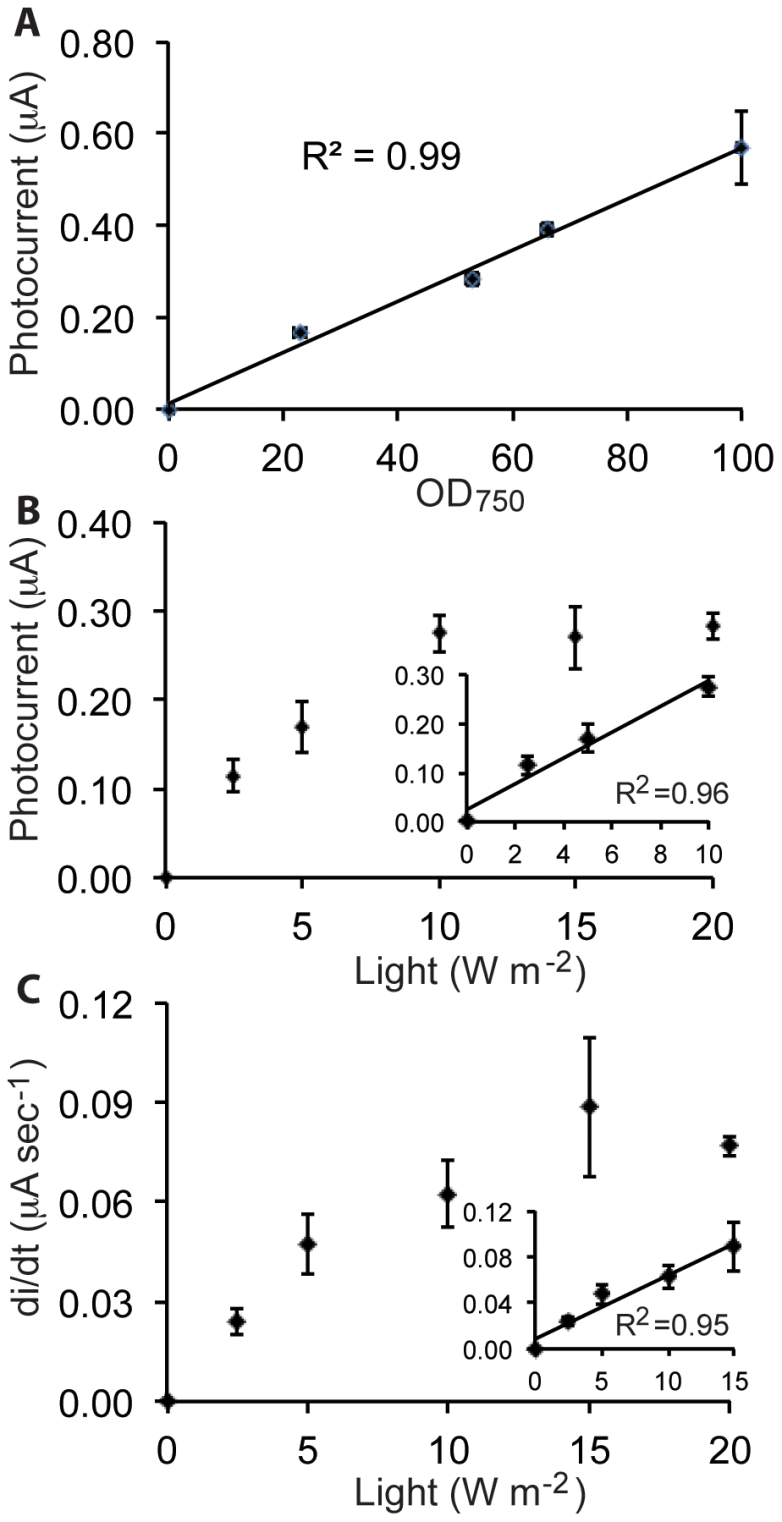
Quantitative analysis of key parameters affecting photocurrent generation. Dependence of photocurrent on cell density (A) and light intensity (B). Part (C) shows the effect of light intensity on the initial rate of increase in photocurrent upon illumination. The inset panels in parts (B) and (C) show only the linear portions of the curves. For (B–C) a cell density of ∼35–50 was used. In A and C, illumination was at a fixed intensity of 20 W m^−2^. The correlation coefficient (R^2^) is given to indicate the linearity of relationships, and error bars represent one standard deviation from the mean of at least 2 independent experiments. Lines show the best linear fit to the data.

Figure 3A shows that for solutions with OD_750_ values in the range 0–100, increasing cell density resulted in increased photocurrent. The relationship is linear with an excellent correlation coefficient (R^2^) of 0.99. For optical densities greater than 100, the linear trend no longer holds, and electrochemical measurements were no longer reproducible. We hypothesize that at higher cell densities the electrode surface may become saturated such that additional direct interactions between the electrode surface and cyanobacterial cells may not be geometrically possible or stable, or light becomes limiting due to self-shading. An approximate geometric calculation suggests that this hypothesis is not implausible. Using the relation that an OD_750_ of 1 corresponds to 1.6×10^8^ cells mL^−1^ and 0.6 mL of cell solution with OD_750_ of 100 led to maximal observed photocurrent, 9.6×10^9^ cells were applied to the electrode experiments with maximal currents. Assuming *Synechocystis* cells of a uniform 1 µm diameter packed into a square array on the electrode surface, a maximum of 3×10^8^ cells would fit onto the electrode. Considering the electrode also has a depth dimension, *i.e.* it is three-dimensional and the depth is neglected in this estimate, and 100% of applied cells are not likely to be in electrical contact with the electrode, these two numbers are in relatively good agreement. In support of this hypothesis we note that at cell densities higher than 100, the electrolyte solution was visibly green suggesting that cells detach from the carbon cloth electrode during the course of the experiment.

Figure 3B shows the impact of light intensity in the range of 0–20 W m^−2^ (0–110 µmol photons m^−2^ s^−1^) on photocurrent using a cell solution of OD_750_ = 50 for application to the electrode. This value was chosen because, as described above, it is in the middle of the linear range for photocurrent production. A positive correlation was observed between light intensity and photocurrent. The relationship was linear (R^2^ = 0.96) up to an intensity of 10 W m^−2^ (equivalent to 55 µmol photons m^−2^ s^−1^). At intensities greater than 10 W m^−2^, a significant increase in photocurrent was not observed. We note that, although perhaps coincidental, this intensity is nearly identical to that under which the cyanobacterial culture was grown, a value chosen because it corresponds to light saturated but not photoinhibited growth for *Synechocystis* [29]. Photocurrent magnitude was not the only parameter directly dependent on light intensity. As shown in Figure 3C, the rate of current increase upon photoillumination (calculated as shown in Figure S3) was also higher at greater light intensities. An approximately linear relationship (R^2^ = 0.96) is observed for light intensities up to 15 W m^−2^; above this intensity the rate remained constant.

Two experiments were undertaken to evaluate whether microbially synthesized or exogenously added redox mediators could facilitate enhanced photocurrent production. First, an electrochemical experiment was performed in which the medium used to grow the cyanobacterial cells was used as the experimental solution instead of fresh BG11 media. No significant increase in photocurrent was observed with spent medium relative to the experiment in fresh medium (wild type 8.49±0.25 nA/OD_750_; wild type in spent medium 7.45±0.91 nA/OD_750_). This indicates that no component exclusively present in the used medium, i.e. not present in the fresh medium, served as a redox mediator. Thus, under the growth conditions employed in these experiments, *Synechocystis* does not release stable, extracellular redox mediators into the growth medium that can donate electrons to the electrode. In the second experiment, 5 mM potassium ferricyanide was added as an exogenous redox mediator to provide a possibly more efficient mechanism to shuttle electrons between the cells and the electrode. To ensure that reduced ferrocyanide produced by the immobilized cells could be efficiently re-oxidized by the working electrode, a higher electrochemical potential bias of +497 mV *vs.* SHE was employed for this experiment. Nonetheless, again significantly enhanced photocurrent was not observed relative to the unmediated experiments (wild type:8.49±0.25 nA/OD_750_; wild type+ferricyanide: 8.2±1.1 nA/OD_750_).

### Strains lacking photosystem II have significantly diminished capacity to produce photocurrent

**Figure 4:**
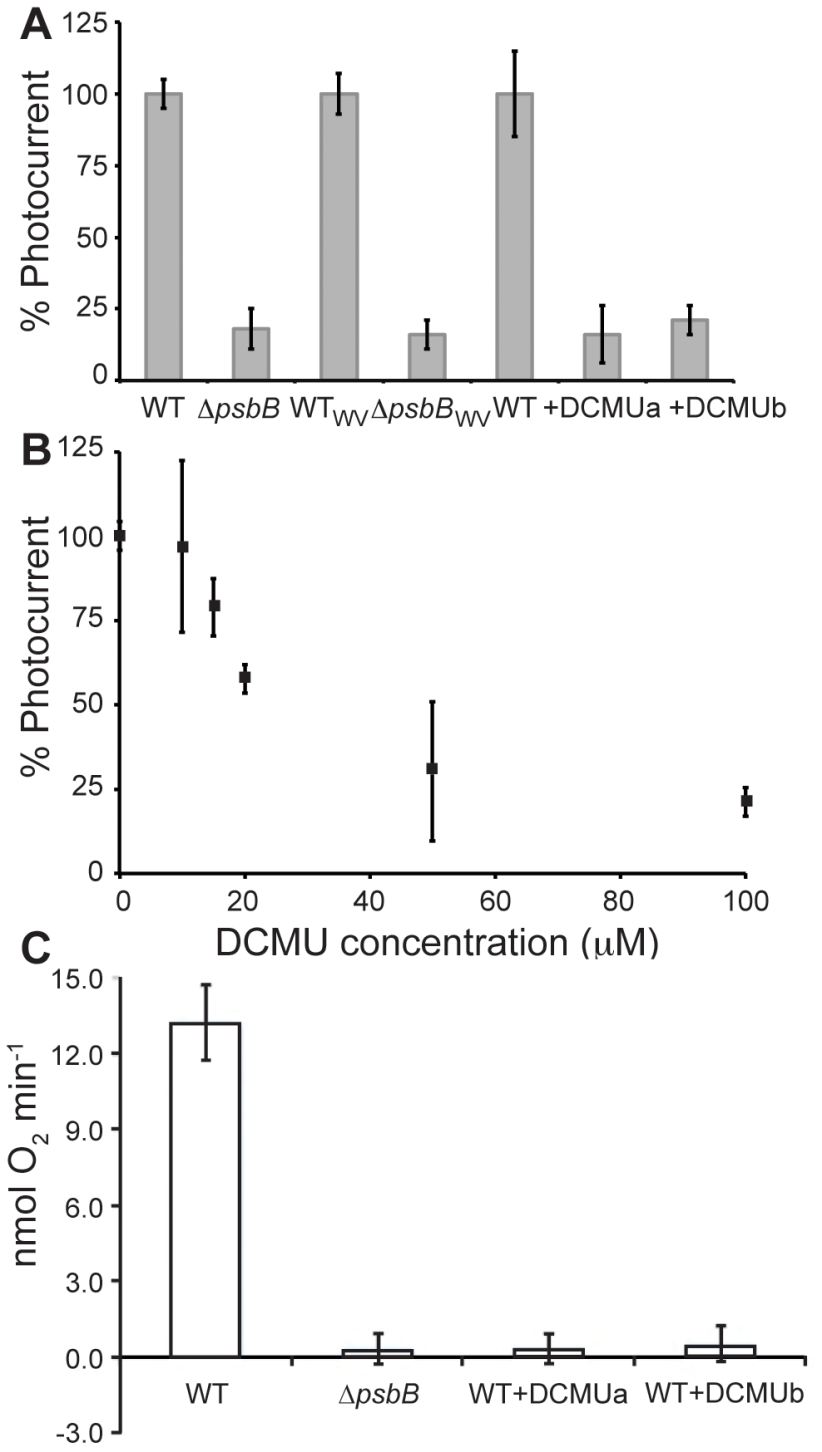
Comparison of photocurrent produced (A, B) and oxygen evolved (C) by wild type or photosynthetically inhibited *Synechocystis* cells. (A) Photocurrent from wild type, Δ*psbB*, Δ*psbB*_WV_, wild type with 100 µM DCMU in solution (+DCMUa), or wild type mixed with 10 µM DCMU during application to the electrode (+DCMUb). Photocurrent is normalized to the cell density of the sample applied to the working electrode, and wild type photocurrent for each experiment is set at 100%. Strains were grown under photomixotrophic (wild type versus Δ*psbB*) or photoautotrophic (± DCMU) conditions (as described in experimental procedures) and harvested at a similar phase of growth (determined by OD_750_). (B) Inhibition of photocurrent in response to DCMU concentration. Photocurrent produced from wild type cells was measured following 5 min dark incubation in the presence of the indicated concentration of DCMU. Appropriate volumes of DCMU were added from a 10 mM stock. The percentage of photocurrent compared to the uninhibited value in the absence of DCMU (100%) is shown. For panels A and B, error bars represent the standard deviation from the mean of at least 2 independent experiments. (C) Oxygen evolution by wild type, *ΔpsbB*, and wild type with 10 µM DCMU *Synechocystis* cells. Data are the average of at least three biological replicates, and the error bars show the standard deviation from the mean. Rates are normalized to OD_750_. For DCMUa, the inhibitor was added directly to the oxygen electrode chamber, and for DCMUb cells were pre-incubated with the inhibitor for five min prior to addition to the measurement chamber.

To determine the role of photosynthetic electron transport in production of photocurrent by *Synechocystis*, electrochemical experiments were undertaken with Δ*psbB* strains. The chlorophyll-binding CP47 protein, encoded by *psbB* (*slr0906*), is required for primary photochemistry in the Photosystem II (PSII) reaction center [30]. The *psbB* open reading frame was replaced with a zeocin (Δ*psbB*; this study, Figure S4) or streptomycin (Δ*psbB*_WV_) [31] resistance cassette. A full description of the production of the Δ*psbB* strain can be found in the experimental methods (Figure S4). Successful deletion of *psbB* was confirmed genotypically by PCR (Figure S4) and phenotypically by the absence of light-dependent oxygen evolution (Figure 4C), photoautotrophic growth, and PSII-associated chlorophyll fluorescence (data not shown). Since the Δ*psbB* mutants cannot grow photoautotrophically, they, and control wild type cultures for comparison, were grown photomixotrophically as described in the experimental section. Cells were harvested in stationary phase (OD_750_ = 2.0) and used in electrochemical experiments as described above.

As shown in Figure 4A, both Δ*psbB* mutants showed an 81–84% reduction of photocurrent relative to the isogenic wild type strain. As an alternative approach to manipulate the photosynthetic electron flux, photocurrent in wild type cells in which electron flow from PSII was blocked by the inhibitor DCMU was also measured. Addition of 100 µM DCMU to the electrochemical cell for five min prior to measurement also resulted in an 84% decrease in photocurrent (Figure 4A, +DCMUa). Lower concentrations of DCMU resulted in a dose-dependent reduction of photocurrent (Figure 4B). Alternatively, application of a cell paste premixed with only 10 µM DCMU to the electrode, resulted in a similar inhibition of photocurrent (Figure 4A, +DCMUb). It is likely that since the electrolyte solution in the electrochemical cell is not stirred, a higher DCMU concentration is necessary to achieve inhibition than if the cells and inhibitor were confined in a smaller volume. Controls in which the same volumes of dimethyl sulfoxide (DMSO, the solvent for the DCMU) were added to the electrolyte did not reduce photocurrent (wild type 8.49±0.25 nA/OD_750_; wild type +DMSO 8.16±1.2 nA/OD_750_).

## Discussion

This work reports the use of a simple, mediatorless bioelectrochemical system to rapidly measure photocurrent produced by *Synechocystis* sp. PCC6803 without the need to formally grow a mature biofilm on an electrode surface. It is the first time that a system that does not rely on a mediator or biofilm grown over multiple hours or days has been exploited to gain a mechanistic understanding of EET. Using planktonically grown cells speeds the process of obtaining biomass so that experimental throughput is significantly enhanced relative to systems employing growth as a biofilm. Furthermore, the absence of chemical redox mediators is advantageous since it allows this approach to be used to explore the native mechanisms of extracellular electron transfer by cyanobacteria. In contrast, chemical redox mediators may accept electrons simultaneously from a number of intracellular components, effectively short circuiting the natural, extracellular wiring of the cells [28]. With that in mind, the mediatorless system described here represents an important new bioelectrochemical tool to elucidate the inherent biochemical circuitry of EET in phototrophic microorganisms and may generate information useful for scaling up this phenomenon for industrial applications.

In our mediatorless system, planktonically grown *Synechocystis* cells produced light-dependent photocurrent. We showed that photocurrent was strictly dependent on the quantity of live cyanobacterial cells in the electrochemical experiment and the intensity of the light, indicating that it is a biological and photosynthetically driven process. Furthermore, as shown by others [11], the rate at which photocurrent increased was also dependent on light intensity (Figure 3C). Thus the electrochemical data is responsive to cellular physiology and can be used to probe the mechanism of EET. Anode respiring bacteria have been shown to employ two distinct types of mechanisms to exchange electrons with extracellular substrates: direct and indirect. *Shewanella* sp. can synthesize and secrete flavins, effectively producing their own redox mediators for indirect transfer of electrons between the cell surfaces and the electrode [32]. In this situation, using supernatants from cell cultures, as opposed to fresh solution, in electrochemical experiments results in substantially increased current production [17], and flavins can accumulate up to micromolar concentrations [33]. No evidence for flavin secretion by *Synechocystis* has been previously reported. Utilizing the media in which the *Synechocystis* cells were grown as the solution for the electrochemical experiments did not enhance photocurrent production. Furthermore, addition of the exogenous mediator ferricyanide did not impact our results. Thus we conclude that, in these experiments, *Synechocystis* is in direct electronic contact with the carbon electrode and does not utilize either endogenously produced mediators or the artificial redox shuttle ferricyanide to transfer electrons. This is similar to reports from *Geobacter* sp. [34], anode respiring bacteria that are thought to employ bacterial nanowires to facilitate direct electron exchange between the microroganism and the electrode surface. It is possible that such nanowires would be impossible to detect at the resolution of our SEM experiments. Nonetheless, we note that SEM images of our microbial electrodes (Figure 1B) show cells apparently in direct contact with the carbon surface. Thus, although it suggests that in our hands the exchange of electrons between *Synechocystsis* and the electrode is direct, the cellular components responsible for this transfer remain to be determined.

In comparing this work to previous reports of bioelectrochemical devices employing anode respiring bacteria such as *Shewanella* or *Geobacter* sp., it is interesting to consider the current produced per cell. Although currents on the order of 100 fA cell^−1^ have been reported for anode respiring bacteria [35], photocurrents in this system are much lower. Assuming all cyanobacterial cells introduced into the experiment are detectable, we can place a lower limit on *Synechocystis* photocurrent production of 0.08 fA cell^−1^. As described in the results, geometric arguments and the SEM images suggest that this assumption is inaccurate and likely overestimates the microbial cells by at least an order of magnitude. Nonetheless, the dramatically lower current produced by *Synechocystis* is no doubt related to the fact that cyanobacteria possesses a number of metabolic pathways that compete with the electrode for electrons. Thus extracellular photocurrent is likely attributable to only a very small fraction of the total electron flux through the organism and may correspond to a valve mechanism to release excess reducing equivalents in extreme conditions.

Previous work has provided hints to the physiological source of photocurrent production by cyanobacteria [11], [36]. However, the investigation of the Δ*psbB* mutants reported herein is the first use of a genetically modified organism affected in photosynthetic electron transport in a BPV to demonstrate conclusively that the majority of photocurrent generated by *Synechocystis* is directly attributable to electrons derived from water splitting by PSII. This corroborates previous experiments reported by others with inhibitors as well as the observation that green light was not able to drive photocurrent production [9], [11], [36]. Bombelli and co-workers showed that although DCMU caused 96% inhibition of oxygen evolution by PSII, only 63% of photocurrent was inhibited. The authors suggested that the residual current in the presence of DCMU could be attributable to respiratory electron transport [11]. Our results bolster this hypothesis. We note that the residual photocurrent produced by the Δ*psbB* mutants (16–19% of full photocurrent) is nearly identical to that measured for wild type cells in the presence of 100 µM DCMU (16%) or following premixing of cells with 10 µM DCMU before application to the electrode (21%). This concentration of DCMU completely prevented oxygen evolution in cell samples of equivalent density to those applied to the electrode (Figure 4C) and resulted in an almost complete absence of PSII-associated variable fluorescence (data not shown). Experiments with DCMU in a mutant deficient in succinate and NADPH dehydrogenases, the experimentally confirmed major respiratory electron donors in *Synechocystis*, will be necessary to conclusively demonstrate that these processes are responsible for the PSII-independent photocurrent.

Work is currently underway to use the system described here to screen photocurrent generated from mutant strains in which additional hypothesized components of the electrogenic pathway have been deleted. As demonstrated by our preliminary studies of the Δ*psbB* strains, normalization of photocurrent to account for cell density, chlorophyll concentration and oxygen evolution rate will allow direct, robust, quantitative comparison between strains. This will make it possible to confirm that observed differences are a result of interruption of the native electron transfer pathway as opposed to alteration of the rate of water splitting by PSII. Such mechanistic experiments are only made possible by using our mediatorless system, since the presence of membrane-permeable electron shuttles may complicate interpretation of results. In principle, it should now be possible to identify the complete electron transfer pathway from the thylakoid membrane to the cell surface and the electrode beyond. Such knowledge will likely prove invaluable to synthetic biologists as they seek to engineer phototrophs capable of producing higher photocurrents.

## Materials and Methods

### Biological material and growth conditions

All bacterial strains used in this study are detailed in Table 1. A glucose-tolerant (GT) strain of *Synechocystis* sp. PCC 6803 (provided by Prof. Peter Nixon, Imperial College London) [37] was used as the wild type (WT), and a Δ*psbB* deletion mutant was generated in this strain background. Additional, independent GT wild type (WT_WV_) and Δ*psbB* (Δ*psbB*_WV_) strains were provided by the laboratory of Prof. Wim Vermaas (Arizona State University). *Synechocystis* was cultured in BG11 media [38] under photoautotrophic or photomixotrophic conditions [39]. For photoautotrophic growth, 200 ml cultures contained within 250 ml flasks were sparged with sterile air at 30°C under a constant illumination of approximately 50 µmol photons m^−2^ s^−1^. For photomixotrophic growth, 5 mM glucose was added to the medium. Growth was monitored by measurement of the optical density at 750 nm (OD_750_). For growth on plates, BG11 was supplemented with 10 mM TES(N-[tris(hydroxymethyl)methyl]-2-aminoethanesulfonic acid)-KOH pH 8.2, 1.5% (w/v) agar, 0.3% (w/v) sodium thiosulphate, 5 mM glucose, and antibiotics as indicated.

**Table 1:** *Synechocystis* sp. PCC 6803 strains used in this study.

Strain	Details	Source
WT	Glucose tolerant strain of *Synechocystis* sp. PCC6803 [37].	Peter Nixon (Imperial College London)
Δ*psbB*	*psbB* deletion mutant. Nucleotides 31–1417 of the *psbB* (*slr0906*) open reading frame replaced with a zeocin resistance cassette.	This study
WT_WV_	Glucose tolerant strain of *Synechocystis* sp. PCC6803 [37].	Wim Vermaas (Arizona State University)
Δ*psbB*_WV_	*psbB* deletion mutant described by Howitt et al. Resistant to streptomycin [31].	Wim Vermaas (Arizona State University)

### Electrochemical measurements

Electrochemical measurements were performed using a CHI 1200A potentiostat (CH instruments, Inc. Austin, Texas). A single chamber glass electrochemical cell containing 10 ml of BG11 media as the electrolyte was employed with a saturated Ag/AgCl reference electrode (CH instruments, Inc. Austin, Texas) and a platinum wire as the counter electrode (Figure 1A). The working electrode consisted of a 3×1 cm piece of carbon cloth (1K plain weave ultralight carbon fibre fabric, 0.009” thick, Fibre Glast Developments Corporation, Brookville, Ohio). Illumination was provided by an LH7 red LED light source (peak λ = 660 nm; Hansatech, Kings Lynn, UK). For electrochemical analysis, cells were harvested at the desired phase of growth (determined from OD_750_) by centrifugation (3,500× g, 22°C, 30 min), and the pellets were washed with and resuspended in fresh BG11 to an OD_750_ of approximately 100. To immobilize cells on the working electrode, resuspended cells (0.1–0.6 ml) were mixed with BG11 (0–0.5 ml) to a total volume of 0.6 ml and were evenly applied to the electrode surface and allowed to dry for approximately 120 min or until the cells adhere but still maintain a green sheen. A photograph of a cell coated electrode can be found in Figure S1. The cell density applied to the electrode is expressed as OD_750_. For conversion of cell densities to numbers of cells, we have used the relationship OD_750_ = 1 (a.u) corresponding to 1.6×10^8^ cells mL^−1^ [29]. All electrochemical experiments were undertaken at room temperature (*circa* 22°C) with an applied potential of +237 mV *vs.* SHE, unless otherwise stated.

### SEM analysis

Samples were fixed in 50 mM sodium phosphate buffer (pH 7.2) with 2% glutaraldehyde for 30 min at room temperature and then washed three times in the same buffer for a total of 30 min. After a second fixation step for 30 min at room temperature in the same buffer plus 0.5% osmium tetroxide, samples were washed three times with deionised H_2_O. Samples were critical point dried with carbon dioxide (Balzers CPD020 unit), mounted on AI specimen stubs, and coated with approximately 15 nm of gold palladium (Technics Hummer-II sputter-coater). Sample analysis was performed with a JEOL JSM-6300 SEM operated at 15 kV, and images were acquired with an IXRF Systems digital scanning unit.

### Deletion of *psbB*

PCR was performed using Accuzyme DNA polymerase (Bioline, London, UK) and oligonucleotide primers were purchased from biomers.net. A *Synechocystis* Δ*psbB* (*slr0906*) deletion mutant was generated by replacing nucleotides 31–1417 (of the 1524 bp open reading frame) with a zeocin resistance cassette isolated from plasmid pZeo (Invitrogen, Paisley, UK) using primers p7 and p8 (see Table 2 for primer sequences). PCR was used to amplify the upstream and downstream flanking regions of *psbB* from wild type *Synechocystis* genomic DNA using primer pairs p1–p2 and p3–p4. Primers p2 and p3 added extensions with sequence homology to the zeocin cassette to the flank PCR products such that when they were mixed with the cassette in an equimolar ratio in a further PCR, primer pair p1–p4 amplified a product in which the cassette was inserted between the upstream and downstream flanks. Transformation of *Synechocystis* with the resulting linear constructs was performed as descrbed by Williams [37]. Selection and segregation of genome copies was achieved by repeated streaking on BG11 plates with increasing zeocin concentration (2.5–20 µg/ml). Transformants homozygous for the deletion were verified by PCR amplification using template DNA derived from wild type or mutant cells (Figure S4). The independently generated Δ*psbB* mutant with the gene replaced by a streptomycin resistant cassette [31] is referred to as Δ*psbB*_WV_ to avoid confusion with the strain generated in the current work.

**Table 2:** Oligonucleotide primers used in this study^a^.

Primer	Sequence (5′ to 3′)
p1	GAATCCGCACTTTGGAGTAT
p2	*ACATTAATTGCGTTGCGCTCACTGC* TGTATGAACGCGATACCAAG
p3	*CAACTTAATCGCCTTGCAGCACAT* CTCTGTTCCGTGACGTATTT
p4	TCATAGCACACTGGTAAGGT
p5	CTGCTGCCCACATCGTTCTA
p6	GGTGGCATTACCGTACCACA
p7	GCAGTGAGCGCAACGCAATTAATGT
p8	ATGTGCTGCAAGGCGATTAAGTTG

a For primers p2 and p3 the italicised sequence is homologous to the 5′ (p2) or 3′ (p3) end of the zeocin resistance cassette.

### Assessment of photosynthetic performance

The rate of oxygen consumption/evolution from whole cells was measured in BG-11 medium at room temperature using an Oxylab meter fitted with a DW1 liquid-phase chamber (Hansatech, Kings Lynn, UK). Following determination of oxygen consumption in the dark, the rate of photosynthesis was measured by illumination in the presence of 10 mM NaHCO_3_. The apparent PSII photochemical quantum efficiency (Fv/Fm) defined by Kolber *et al.* [40] was measured using a FASTtacka™ Mk II Fast Repetition Rate fluorometer integrated with a FASTact™ Laboratory system (Chelsea Technologies Group Ltd, Surrey, UK). Where indicated, DCMU was added to a final concentration of 10–100 µM to block electron flow from PSII. All measurements were standardized using OD_750_.

## Supporting Information

Figure S1 **Photograph of cloth electrode following application of cells for two hours.**
(TIF)

Figure S2 **Electrical response of a bare carbon cloth electrode exposed to a dark/light cycle.**
(TIF)

Figure S3 **Calculation of the initial rate of increase in photocurrent.** (**A**) The magnitude of photocurrent was measured upon illumination with red light over a range of intensities. For clarity just two examples, 5 and 15 W m^−2^ are shown. To determine if there was any difference in the initial rate at which current increased upon illumination with increasing light intensity, the first 20 seconds (shown by the dotted box) of the increase was analyzed, as shown in part (**B**). A linear regression was fitted to each data set, and the slope of each line was calculated as a measure of the rate of increase in current per second, as presented in Figure 3C of the paper.
(TIF)

Figure S4 **Scheme for deletion of *psbB*.** (A) Strategy for replacement of *psbB* (*slr0906*) with the zeocin resistance cassette (Zeo^R^) by splicing overlap extension PCR. Primer pairs p1–p2 or p3–p4 were used to amplify an ∼400 bp fragment of the DNA upstream or downstream of the *psbB* locus; primers p2 and p3 contained sequence homology to the 5′ or 3′ end of Zeo^R^ respectively. When the three fragments were mixed in a subsequent PCR, single complementary strands annealed and primer pair p1–p4 amplified the full-length deletion construct. This construction was introduced into *Synechocytsis* sp. PCC 6803 by natural transformation, and transformants were segregated on zeocin-containing plates. (B) The wild type *psbB* gene and flanking DNA. (C) The same region in Δ*psbB* transformants, in which Zeo^R^ has replaced the *psbB* gene. In (B) and (C) the positions of primer annealing and the approximate sizes of PCR products generated during transformant screening are shown. (D) Agarose gel analysis of PCR amplicons confirming Δ*psbB* is homozygous for the deletion allele at the *psbB* locus. Lanes 1, 3 and 5 show PCR products amplified using template DNA from wild type and lanes 2, 4, 6 and 7 from Δ*psbB*. The primer pair used in each reaction is indicated above the gel. Lane M = HyperLadder™ I molecular weight marker (Bioline, London, UK).
(TIF)
