# Supplementary figures and images for: A Bioelectrochemical Approach to Characterize Extracellular Electron Transfer by Synechocystis sp. PCC6803
Source: PLoS One. 2014 Mar 17;9(3):e91484. doi: 10.1371/journal.pone.0091484 (PMC3956611; doi:10.1371/journal.pone.0091484)

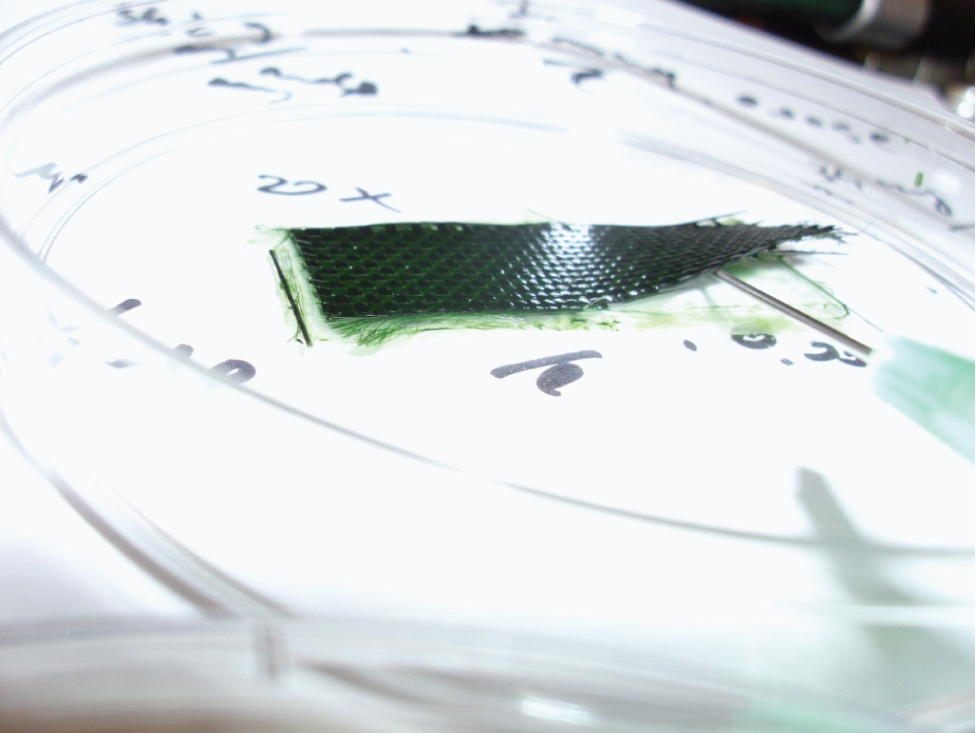

Supplement: Figure S1 — Photograph of cloth electrode following application of cells for two hours. (TIF) [file pone.0091484.s001.tif]

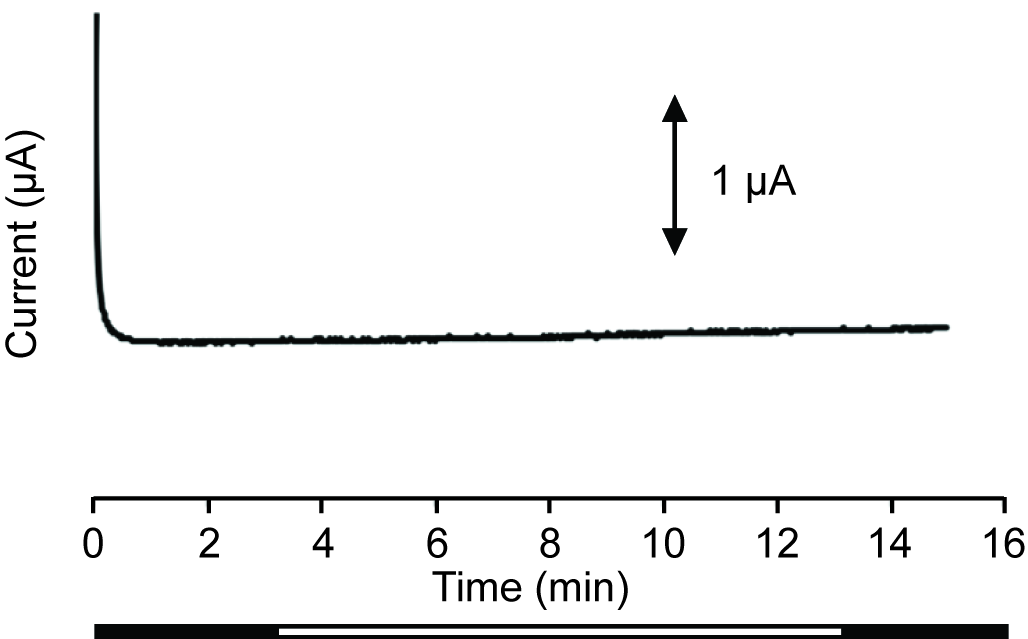

Supplement: Figure S2 — Electrical response of a bare carbon cloth electrode exposed to a dark/light cycle. (TIF) [file pone.0091484.s002.tif]

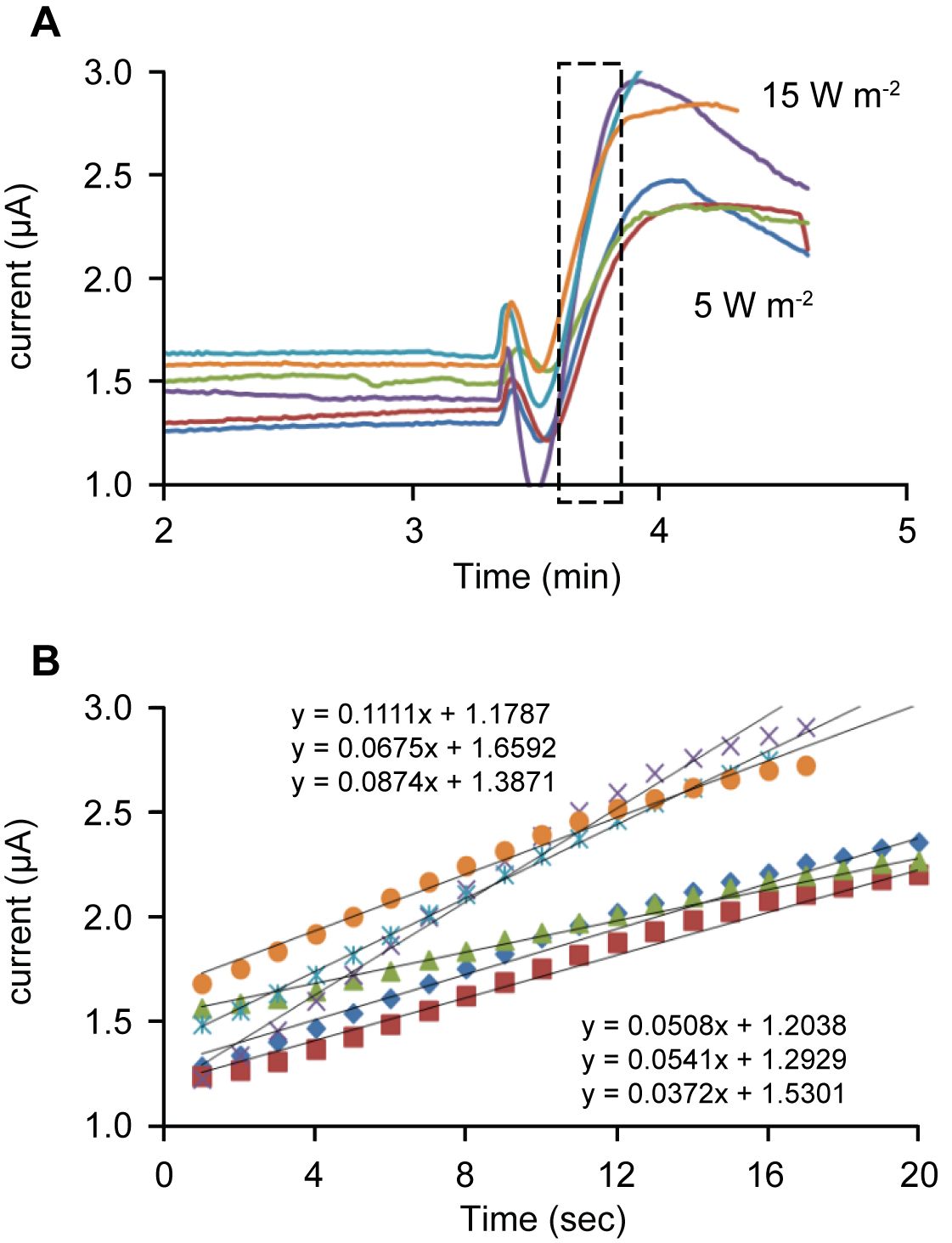

Supplement: Figure S3 — Calculation of the initial rate of increase in photocurrent. (A) The magnitude of photocurrent was measured upon illumination with red light over a range of intensities. For clarity just two examples, 5 and 15 W m−2 are shown. To determine if there was any difference in the initial rate at which current increased upon illumination with increasing light intensity, the first 20 seconds (shown by the dotted box) of the increase was analyzed, as shown in part (B). A linear regression was fitted to each data set, and the slope of each line was calculated as a measure of the rate of increase in current per second, as presented in Figure 3C of the paper. (TIF) [file pone.0091484.s003.tif]

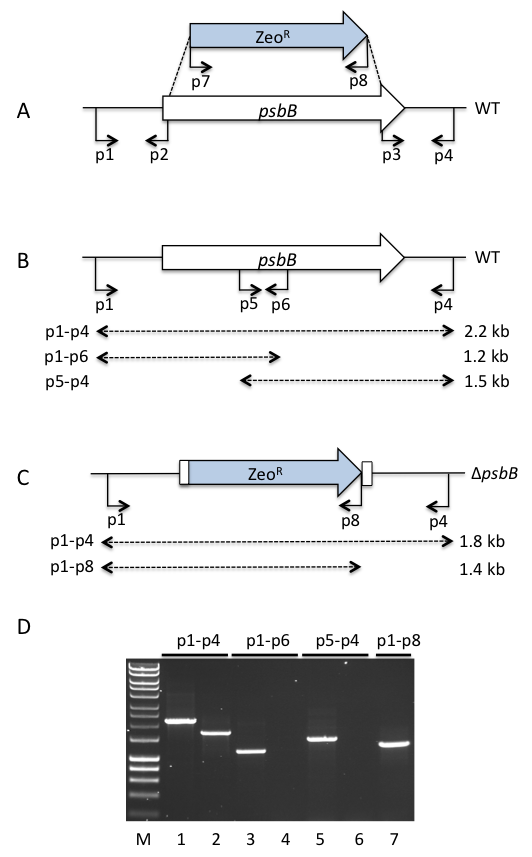

Supplement: Figure S4 — Scheme for deletion of psbB. (A) Strategy for replacement of psbB (slr0906) with the zeocin resistance cassette (ZeoR) by splicing overlap extension PCR. Primer pairs p1–p2 or p3–p4 were used to amplify an ∼400 bp fragment of the DNA upstream or downstream of the psbB locus; primers p2 and p3 contained sequence homology to the 5′ or 3′ end of ZeoR respectively. When the three fragments were mixed in a subsequent PCR, single complementary strands annealed and primer pair p1–p4 amplified the full-length deletion construct. This construction was introduced into Synechocytsis sp. PCC 6803 by natural transformation, and transformants were segregated on zeocin-containing plates. (B) The wild type psbB gene and flanking DNA. (C) The same region in ΔpsbB transformants, in which ZeoR has replaced the psbB gene. In (B) and (C) the positions of primer annealing and the approximate sizes of PCR products generated during transformant screening are shown. (D) Agarose gel analysis of PCR amplicons confirming ΔpsbB is homozygous for the deletion allele at the psbB locus. Lanes 1, 3 and 5 show PCR products amplified using template DNA from wild type and lanes 2, 4, 6 and 7 from ΔpsbB. The primer pair used in each reaction is indicated above the gel. Lane M = HyperLadder™ I molecular weight marker (Bioline, London, UK). (TIF) [file pone.0091484.s004.tif]
